# Advances in Hard–to–Cut Materials: Manufacturing, Properties, Process Mechanics and Evaluation of Surface Integrity

**DOI:** 10.3390/ma13030612

**Published:** 2020-01-30

**Authors:** Szymon Wojciechowski, Grzegorz M. Królczyk, Radosław W. Maruda

**Affiliations:** 1 Faculty of Mechanical Engineering and Management, Poznan University of Technology, 3 Piotrowo St., 60-965 Poznan, Poland; 2 Department of Manufacturing Engineering and Production Automation, Faculty of Mechanical Engineering, Opole University of Technology, 5 Mikolajczyka Street, 45-271 Opole, Poland; g.krolczyk@po.opole.pl; 3 Faculty of Mechanical Engineering, University of Zielona Gora, Prof. Z. Szafrana Street 4, 65-516 Zielona Gora, Poland; r.maruda@ibem.uz.zgora.pl

**Keywords:** hard–to–cut materials, machining, additive manufacturing, mechanics, surface integrity

## Abstract

The rapid growth of a modern industry results in a growing demand for construction materials with excellent operational properties. However, the improved features of these materials can significantly hinder their manufacturing, therefore they can be defined as hard–to–cut. The main difficulties during the manufacturing/processing of hard–to–cut materials are attributed to their high hardness and abrasion resistance, high strength at room or elevated temperatures, increased thermal conductivity, as well as their resistance to oxidation and corrosion. Nowadays the group of hard–to–cut materials includes the metallic materials, composites, as well as ceramics. This special issue, “Advances in Hard–to–Cut Materials: Manufacturing, Properties, Process Mechanics and Evaluation of Surface Integrity” provides a collection of research papers regarding the various problems correlated with hard–to–cut materials. The analysis of these studies reveals primary directions regarding the developments in manufacturing methods, and the characterization and optimization of hard–to–cut materials.

Nowadays, in many industrial branches, the growing demand for construction materials with excellent operational and mechanical properties is observed. Especially in the aerospace, biomedical, electronic and automotive industries, construction materials with high hardness, abrasion resistance, a high strength in a range of various temperatures, increased thermal conductivity, as well as resistance to oxidation and corrosion, are very often employed. Unfortunately, these unique features significantly deteriorate the machinability of these materials, and thus they are defined as hard–to–cut.

The major problems occurring during the machining of hard–to–cut materials include the high values of cutting forces, high levels of vibrations in machining systems, the concentration of heat, the growth of cutting temperature, rapid tool wear and the risk of catastrophic tool failure, as well as frequent stability loss and a significant deterioration in surface finish.

The group of hard–to–cut materials is extensive and still expanding, attributed to the development of novel manufacturing techniques (e.g., additive technologies). Currently, the group of hard–to–cut materials includes hardened and stainless steels, titanium, cobalt and nickel alloys, composites and ceramics, as well as the hard clads fabricated by additive techniques.

This special issue, “Advances in Hard–to–Cut Materials: Manufacturing, Properties, Process Mechanics and Evaluation of Surface Integrity” provides the collection of thirteen research articles presenting recent activity and developments in this field. Studying these works reveals the current problems and research directions concerning hard–to–cut materials. Among these, the novel production and machining techniques and the production/machining optimization methods, as well as the novel measurement/characterization techniques, can be identified (Figure 1).

The problems regarding the application of novel manufacturing techniques for hard–to–cut materials are presented in four papers. Kluczyński et al. [1], investigated the porosity and the microhardness of 316L austenitic steel, manufactured with the application of selective laser melting (SLM) additive technology. The authors have revealed that microstructure porosity is affected by the hatching distance and exposure velocity. As the hatching distance increases, the microstructure porosity of this element increases, and the decrease in exposure velocity causes a decline in porosity level. Moreover, an increase in microhardness with an increase in the exposure energy density was observed. This observation can be connected with the combined effect of grain refinement strengthening (Hall–Petch relation) and grain boundary strengthening. Prakash et al. [2] developed a method for the production of porous Mg–based biodegradable structures, based on the hybridization of elemental alloying and spark plasma sintering technology. The authors employed suitable proportions of silicon (Si) and hydroxyapatite (HA) to enhance the mechanical, chemical, and geometrical features. They found that the addition of HA and Si elements affects the improvement of structural porosity, with a low elastic modulus and hardness of the Mg–Zn–Mn matrix, respectively. Moreover, the addition of both HA and Si elements refined the grain structure and improved the hardness of the as–fabricated structures. Authors have also detected the formation of various biocompatible phases, whose appearance enhances the corrosion performance and biomechanical integrity of manufactured structures. Guo et al. [3] proposed ultraviolet–curable resin bonding for a precision abrasive machining tool, aiming to deliver a rapid, flexible, economical, and environment–friendly additive manufacturing process to replace the hot press and sintering process. Authors have employed a customized ultraviolet curing system based on the Machine UV–100, and the Dymax 5000 flood ultraviolet curing system used for the initial material properties test of the cured ultraviolet–curable resin composites. The manufactured precision abrasive machining tool consisted of an ultraviolet–curable epoxy resin 425 as a bond and monocrystalline diamond grains as abrasives. Authors have proved that the application of an abrasive machining tool equipped with the ultraviolet–curable resin bonding during lapping process enabled an approximately 10% lower surface roughness parameter *Ra* and 25% less weight loss of the workpiece than those obtained in the iron plate lapping process. Prakash et al. [4], in their study, employed two methods (electric discharge coating (EDC) and electric discharge machining processes (EDM)) to coat a composite layer TiO_2_–TiC–NbO–NbC on the Ti–64 alloy. The conducted research revealed that the application of the EDC process with a high peak current and high Nb–powder concentration enabled the formation of a crack–free thick layer (215 µm) on the workpiece surface. Moreover, further inspections have shown that the obtained coating has a high hardness and adhesion strength, which enables it to enhance the wear resistance of the Ti–64 alloy.

This collection of papers also presents that—apart from the novel manufacturing technologies—the current research direction of hard–to–cut materials involves novel machining techniques. This scientific problem matter is covered in three papers. Khanna et al. [5] employed the ultrasonic–assisted turning (UAT) process of the Nimonic–90 superalloy in order to replace the conventional cutting and obtain improved technological effects. The results showed that the ultrasonic–assisted turning process affects the reduction in surface roughness and power consumption values as compared with the conventional turning process. This is correlated with the micro–chipping effect induced by UAT process kinematics. Besides, the chips formed during the ultrasonic–assisted turning were regular and fragmented when compared to those obtained from the conventional turning process. The ultrasonic–assisted machining has been also applied by Tan et al. [6] to the micro–groove manufacturing in the Ti–6Al–4V alloy. The application of this kind of machining process aims to minimize the level of material swelling and springback and improve the machining quality. The experimental results proved that the material swelling and springback were significantly reduced and the surface integrity was substantially improved during the ultrasonic elliptical vibration–assisted cutting process in comparison to the conventional cutting process. Apart from vibration–assisted cutting, the novel methods of machining related to hard–to–cut materials involve also the application of nano–cutting fluids. Gupta et al. [7] employed different nano–cutting fluids (aluminum oxide (Al_2_O_3_), molybdenum disulfide (MoS_2_), and graphite) during the turning of the Inconel 800 alloy under the minimum quantity lubrication (MQL) conditions. The obtained results reveal that the MoS_2_– and graphite–based nanofluids can affect the improvement in cutting effects, especially at the high cutting speed values. Moreover, the overall performance of graphite–based nanofluids is better in terms of good lubrication and cooling properties. The presence of small quantities of graphite in vegetable oil significantly improves the machining characteristics of Inconel–800 alloy as compared with the two other nanofluids.

The next important research direction regarding the hard–to–cut materials includes the production/machining optimization methods. These problems are covered in four research papers. Singh et al. [8] applied the Vashy–Buckingham π–theorem for the selection of input parameters of the fused deposition modeling assisted by the investment casting process, enabling the obtainment of optimal hardness, dimensional accuracy, and surface roughness of manufactured aluminum matrix composite (AMC). The validation of the proposed models, conducted on the basis of the ANOVA method, proves their applicability to the optimization of aluminum matrix composite manufacturing during the fused deposition modeling assisted by the investment casting. Buj–Corral et al. [9] employed a central composite design to model the behavior of surface roughness during ball end milling of hot work–hardened tool steel W–Nr, consisting of a two level factorial design with four factors (24 = 16 experiments), and four central points. The conducted studies have shown that the radial depth of the cut was the most relevant factor on *Ra* and *Rt* for both climb and conventional milling. However, the axial depth of cut, cutting speed and feed per tooth have a slight influence on surface roughness within the investigated range. Mia et al. [10] proposed the application of evolutionary–based algorithms (teaching–learning–based optimization and bacterial foraging optimization) for the optimization of the hardened high–carbon steel AISI 1060 turning process. It was found that teaching–learning–based optimization (TLBO) was found to be superior to the bacteria foraging optimization (BFO) in terms of better convergence and a shorter time of computation—hence, the TLBO is recommended during the optimization of hard turning processes. The hardened steel optimization problems were also investigated by Twardowski and Wiciak–Pikuła [11]. They predicted the tool wear during turning of hardened 100Cr6 steel with the application of multilayer perceptron (MLP)–based artificial neural networks. The obtained results show that selection of the number of neurons in the hidden layer and activation function in the hidden and initial layers significantly affect the reliability of tool wear prediction. Alterations in the model structure at the beginning of its formulation help to achieve the assessments at a satisfactory level. Therefore, the artificial neural network with a multilayer perceptron is an effective method for predicting tool condition during the machining of hard–to–cut materials.

Ultimately, the developments in production, machining and optimization techniques regarding the hard–to–cut materials also entail advancements in metrological description and characterization. As part of this subject, the two research papers were published. Uddin et al. [12] applied a multi–dimensional evaluation of hole quality in an Al6061 alloy after drilling. Authors have employed the novel octagonal–ellipse load cell set–up for measurements of feed force and torque. Moreover, the tests also involved SEM analyses of a drill–bits after cutting and measurements of hole diameter errors with the application of a machine tool probe. Bartkowiak and Brown [13] proposed the novel multiscale method for calculating curvature tensors on measured surface topographies of a 6061 T6 alloy. The curvature tensors were calculated as functions of scale, i.e., size, and position from a regular, orthogonal array of measured heights. Moreover, in the derivations, vectors normal to the measured surface were calculated first, then the eigenvalue problem was solved for the curvature tensor. The validity of these methods has been proven by the high consistency of the results with expectations of manufactured surfaces. These expectations included the nature of the curvature and their orientations relative to manufactured features on the surfaces.

The knowledge contained in papers covered in this special issue can be helpful for the efficient selection of manufacturing and characterization methods, as well as the conditions, strategies and types of tools used during the machining of hard–to–cut materials, allowing the improvement of manufacturing performance and economics.

## Figures and Tables

**Figure 1:**
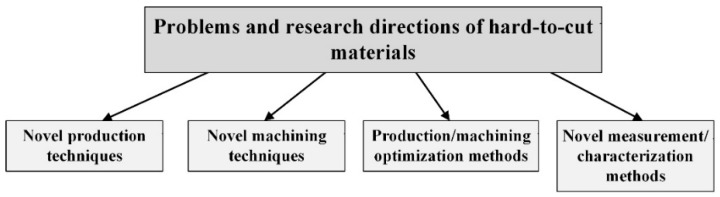
Current major problems and directions concerning the hard–to–cut materials.
